# Bone choristoma of the gingiva: A case report

**DOI:** 10.1016/j.amsu.2021.102474

**Published:** 2021-06-06

**Authors:** Rachid Aloua, Ousmane Belem, Ouassime Kerdoud, Ulrich Opoko, Amine Kaouani, Siham Nagib, Meriem Regragui, Mehdi Karkouri, Tarcissus Konsem, Faiçal Slimani

**Affiliations:** a Faculty of Medicine and Pharmacy, Hassan II University of Casablanca, B.P 5696, Casablanca, Morocco; b Oral and Maxillofacial Surgery Department, CHU Ibn Rochd, B.P 2698, Casablanca, Morocco; c Pathology Department, CHU Ibn Rochd, B.P 2698, Casablanca, Morocco; d Oral and Maxillofacial Surgery Department, CHU Yalgado, Ouedraogo, Ouagadougou, Burkina Faso

**Keywords:** Choristoma, Gum, Treatment

## Abstract

**Introduction:**

Bone choristoma is a benign tumor with normal histology and ectopic location. This paper aims to report a rare case of bone choristoma in the maxillary gingival location.

**Observation:**

The authors report a case of a 39-year-old woman, with a history of maxillary full edentulousness, who consulted for a slight pain evolved for about six months, triggered by movements on the lesion. Clinical examination found growth at the level of the right maxillary gingival alveolar ridge. Surgical biopsy was indicated and performed under local anesthesia. The histological examination of the excisional specimen concluded at a bone choristoma. The patient had a good evolution after the surgical removal.

**Conclusion:**

Choristoma is a rare and benign condition. The management is surgical.

## Highlights

• Choristoma is a rare and benign condition.
• Bone choristoma is a benign tumor that can occur regardless of the patient's age.
• The symptomatology is variable depending on the location and size of the choristoma.
• The treatment is surgical and the absence of recurrence is the rule.

## 1 Introduction

Choristoma is a rare and benign condition. It is a heterotopy consisting of the localization of histologically normal tissue in an ectopic position [1]. In most cases, it is surgically managed [2]. No local recurrence is frequently observed. We report a rare case of bone choristoma at a gingival location.

This case has been reported in line with the SCARE criteria [7].

## 2 Case report

**Fig. 1:**
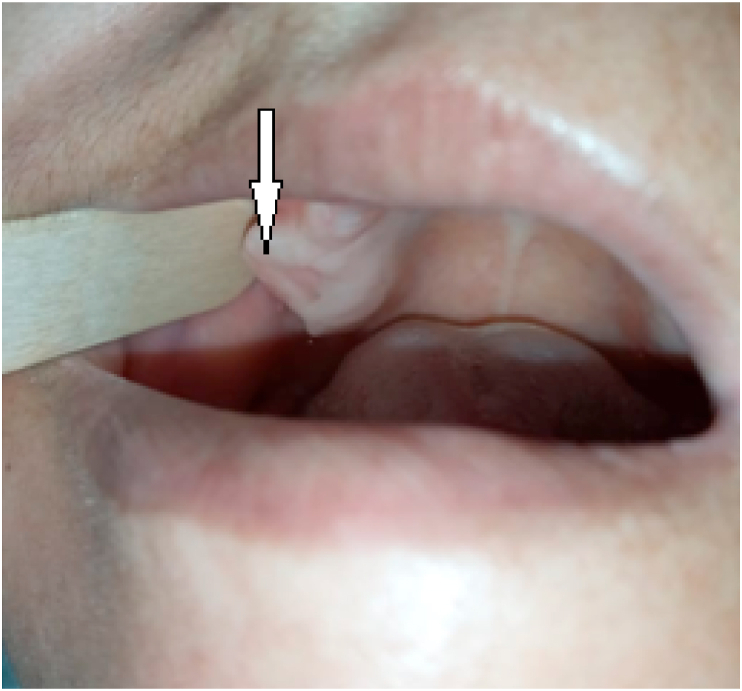
Gingival outgrowth located in the right maxillary attached mucosa.

**Fig. 2:**
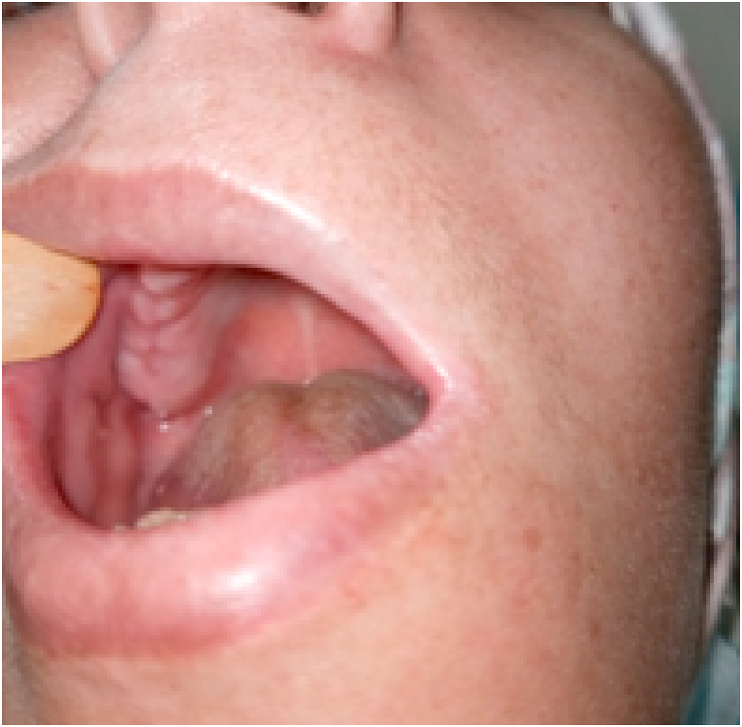
Healing after removal of the gingival growth.

**Fig. 3:**
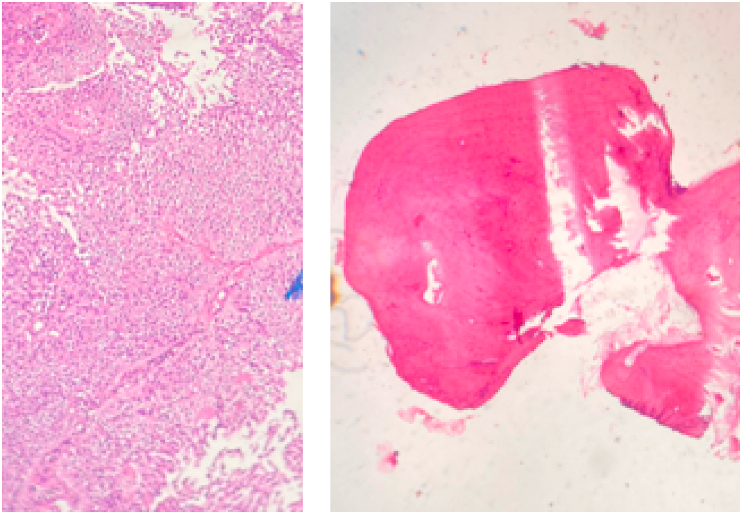
Histological appearance of case composed of dense mature bone, compact and featureless osteocytes, and without prominent osteoblastic border.

A 39-year-old female with a history of maxillary edentulism consulted for moderate pain reported by the patient during masticatory movements. Clinical examination found a right maxillary gingival overgrowth that had been evolving for about six months (Fig. 1). This growth was located on the gingival ridges and protruded vestibular and was troublesome. Tongue movements over the swelling triggered moderate pain. Palpation showed a bony hard swelling attached to the jawbone, the mucosa opposite was of homogeneous white color different from the fibro mucosa. The examination did not find any bleeding on contact or gingival ulceration. The lymph node examination was normal. An excisional biopsy was indicated and performed under local anesthesia allowing removal of the bony growth which was normal in appearance. The mucosa was closed and healing was achieved within a few days (Fig. 2). The patient received analgesic treatment, prophylactic antibiotic therapy, and a mouthwash solution. Pathological examination of the excisional specimen found squamous mucosa with mature bone guts in the chorion. The osteocytes were compact and not very visible. No osteoblastic border was seen. There was no sign of malignancy. The diagnosis of bone choristoma was retained (Fig. 3). The patient was followed up afterwards in the outpatient department at a rhythm of once a month with no sign of recurrence in six months of follow-up.

## 3 Discussion

A bone choristoma is rare and corresponds to a proliferation of normal tissue in an ectopic position [ 1]. Bone choristoma was introduced by Krolls et al. He described a tumor-like growth of mature, normal lamellar bone occurring in the soft tissues of the oral cavity [3]. Bone choristoma is described in both women and men with a female to male ratio of 2.7:1. Bone choristoma can occur at any age [3].In the oral cavity, bronchogenic cyst-type choristoma was also described in the tongue by A. Petraud et al. in a 22-year-old adolescent [4]. An epidermal choristoma occurring on the midline of the gum as a congenital epulis was reported by IzumiYoshioka et al. in a two (02) year old child [5]. In the maxillofacial sphere, bone choristoma was described in the bulbar conjunctiva by A. Martel et al. Salivary gland choristoma in the middle ear was reported by M. S. Boleas-Aguirre et al. [2]. Bone choristoma is asymptomatic in the majority of cases and the clinical signs depend on the location. Signs such as choking, yawning, nausea, and dysphagia are observed [3]. Our patient presented with moderate pain caused by tongue movements and hindering eating.

The CT scan and panoramic radiograph of the maxilla were normal. Histologically, a well-organized central lamellar bone surrounded by periosteum and embedded in fibroadenoma tissue has been described [6]. Osteocytes can be observed in the lacunae of the bone spherule. Osteoblast cell activity at the periphery of the bone mass was discrete [3]. The case we reported presented a squamous mucosa whose chorion was the site of mature bone guts. Osteocytes were also present. The etiology of the bone choristoma is unknown. Ossification of branchial arch remnants and the development of ectopic mesenchymal cells have been discussed [3]. Choristoma of the gum is a benign heterotopy, it can be bony, cartilaginous, or osteocartilaginous or proliferation of other normal tissue in an ectopic position. At the level of the oral cavity can produce following a chronic local irritation in our case the use of dental prosthesis, clinically it presents as a gingival overgrowth that can have a differential diagnosis as peripheral ossifying fibroma, fibroma/fibrosis, with giant cells, epulis of the gum [8]. The treatment was surgical removal [2].

## 4 Conclusion

Bone choristoma is a benign tumor that can occur regardless of the patient's age. The symptomatology is variable depending on the location and size of the choristoma. It is a heterotopia whose diagnosis is histological. The treatment is surgical and the absence of recurrence is the rule.

## 5 Provenance and peer review

Not commissioned, externally peer reviewed.

## Ethical approval

Written informed consent was obtained from the patient for publication of this case report and accompanying images. A copy of the written consent is available for review by the Editor-in-Chief of this journal on request.

## Funding

The authors declared that this study has received no financial support.

## Author statement

Belem Ousmane: Corresponding author writing the paper.

Rachid Aloua: writing the paper.

Ouassime kerdoud: writing the paper.

Savadogo sayouba: writing the paper.

Faiçal Slimani: Correction of the paper.

## Conflicts of interest

Authors of this article have no conflict or competing interests. All of the authors approved the final version of the manuscript.

## Registration of research studies

1. Name of the registry:

2. Unique Identifying number or registration ID:

3. Hyperlink to your specific registration (must be publicly accessible and will be checked):

## Guarantor

Belem Ousmane.

## Consent

Written informed consent was obtained from the patient for publication of this case report and accompanying images. A copy of the written consent is available for review by the Editor-in-Chief of this journal on request.
